# Effects of Caloric Restriction on the Epigenetic Landscape of Hematopoietic Stem Cells

**DOI:** 10.1093/geroni/igab046.1973

**Published:** 2021-12-17

**Authors:** Isabel Beerman

**Affiliations:** NIA, Baltimore, Maryland, United States

## Abstract

During aging, alterations of hematopoietic stem cells are associated with functional decline of the blood system. Caloric restriction (CR) interventions have been reported to improve adult stem cells in other tissue types during aging so we sought to evaluate the effects of CR on the aged HSC compartment. We find significant epigenetic alterations in HSCs isolated from aged mice after life-long CR compared to ad libitum fed aged mice. We further evaluated the epigenetic landscapes and functional potential of aged HSCs shortly after allowing life-long CR mice access to ad libitum food. We uncover epigenetic modification associated with functional alterations of the HSCs, defining potential mechanisms by which restrictions in food consumption affect the aging hematopoietic compartment.

